# Contribution mapping: a method for mapping the contribution of research to enhance its impact

**DOI:** 10.1186/1478-4505-10-21

**Published:** 2012-07-02

**Authors:** Maarten O Kok, Albertine J Schuit

**Affiliations:** 1 Department of Health Sciences and the EMGO Institute for Health and Care Research, VU University, De Boelelaan 1085, Amsterdam, 1081 HV, The Netherlands; 2 National Institute for Public Health and the Environment (RIVM), Antonie van Leeuwenhoeklaan, 9 PO Box 1, Bilthoven, BA, 3720, The Netherlands

## Abstract

**Background:**

At a time of growing emphasis on both the use of research and accountability, it is important for research funders, researchers and other stakeholders to monitor and evaluate the extent to which research contributes to better action for health, and find ways to enhance the likelihood that beneficial contributions are realized. Past attempts to assess research 'impact' struggle with operationalizing 'impact', identifying the users of research and attributing impact to research projects as source. In this article we describe Contribution Mapping, a novel approach to research monitoring and evaluation that aims to assess contributions instead of impacts. The approach focuses on processes and actors and systematically assesses anticipatory efforts that aim to enhance contributions, so-called alignment efforts. The approach is designed to be useful for both accountability purposes and for assisting in better employing research to contribute to better action for health.

**Methods:**

Contribution Mapping is inspired by a perspective from social studies of science on how research and knowledge utilization processes evolve. For each research project that is assessed, a three-phase process map is developed that includes the main actors, activities and alignment efforts during research formulation, production and knowledge extension (e.g. dissemination and utilization). The approach focuses on the actors involved in, or interacting with, a research project (the linked actors) and the most likely influential users, who are referred to as potential key users. In the first stage, the investigators of the assessed project are interviewed to develop a preliminary version of the process map and first estimation of research-related contributions. In the second stage, potential key-users and other informants are interviewed to trace, explore and triangulate possible contributions. In the third stage, the presence and role of alignment efforts is analyzed and the preliminary results are shared with relevant stakeholders for feedback and validation. After inconsistencies are clarified or described, the results are shared with stakeholders for learning, improvement and accountability purposes.

**Conclusion:**

Contribution Mapping provides an interesting alternative to existing methods that aim to assess research impact. The method is expected to be useful for research monitoring, single case studies, comparing multiple cases and indicating how research can better be employed to contribute to better action for health.

## Introduction

Investments in research for health are mostly made with the aim of ultimately contributing to better action for health. The question increasingly being asked is what the benefits are of the tremendous investments in research, and how the beneficial impact can be enhanced [1-5]. Within the health sector, a – albeit fragmented – research domain has emerged that deals with assessing research impact (related terms are: payback, utilization, translation, diffusion, application, implementation, use) [6-11]. Various conceptual frameworks and methods have been developed and, increasingly, empirical studies are reported [6,12-18]. While important progress is being made, the developed methods struggle with operationalizing the dependent variable ‘impact’, attributing impact to individual research projects and identifying the users of research [13]. In addition, the developed methods are primarily used for accountability purposes: showing impact to external funders, instead of learning how to better employ research and enhancing the realization of beneficial contributions.

In this article we will describe *Contribution Mapping*, a novel approach to research monitoring and evaluation that focuses on processes and contributions (instead of products and impacts). While *Contribution Mapping* can be used for accountability purposes, it is especially designed to assist those who seek to better employ research to contribute to action for health. We use the broad term ‘action for health’ as we want to include all activities that are somehow related to health (e.g. contributions to an insurance policy, better practices and technologies, organization of care).

Research does not function as a cannon that shoots knowledge into the world of action, where the targeting and force of the knowledge determines its ‘impact’. The productivity of research for health ultimately lies with the users who have to pick up and combine knowledges (in the plural) and use them for their own purposes, in a complex world full of ongoing processes [19]. The subsequent changes in action achieved are the result of the distributed agency of multiple actors and a confluence of actions, knowledges and circumstances. Such changes are part of evolving, complex and open systems in which change is continuous, non-linear, multi-directional and difficult to control [20]. The consequence is that achieved changes cannot realistically be attributed to a single research project as ‘source’. The utilization of research results is often diffuse: it contributes to one’s view of the world, to signals being taken into account, in addition to the more recognizable production of specific knowledge with an instrumental function [21]. People often do not realize where they get their knowledge from and may not be able to explain what role knowledge played in their behaviour. A related challenge is that the pathways from research to ‘impacts’ are very diverse: sometimes short and traceable, but often long, through multiple reservoirs, and via utilization at untraceable times and places. Though there might be a ‘trail’ from the cowpox vaccination study by Jenner at the end of the 18^th^ century, to the global eradication of smallpox in 1977, it seems impossible to trace it and erroneous to attribute eradication to Jenner’s study as source.

The attribution problem, challenge of identifying users and the diffuseness and diversity of research & action pathways make an assessment of the ultimate ‘impact’ of a research project problematic. Therefore, *Contribution Mapping* focuses on the actors that are involved in, or directly interact, with a research project and aims to assess contributions instead of impacts. A contribution to action refers to the activities which turn a novel combination of knowledges into a ‘going concern’ as a part of practices, a component in successful innovation or an element in decisions and their implementation.

An additional drawback of focusing on ultimate impacts is that this does not provide the kind of information and feedback required by those involved in a research & action pathway to improve their performance. Even if we could trace smallpox eradication in 1977 back to the work of Jenner, that would not indicate what he could have done differently to enhance the use of his results. To use *Contribution Mapping* for learning and improvement purposes, it has to provide stakeholders with actionable knowledge about deliberate efforts that enhance the likelihood that research contributes to action. In order to refer to such efforts, we introduce the notion of *alignment efforts* (alignment emphasizes that accommodation can take place on the side of research and/or on the side of action, instead of a one-way research to action dynamic). Some examples of *alignment efforts* are engaging policymakers in research priority setting, writing dissemination plans and engaging patients in the interpretation of results [22-29]. Such efforts stimulate those involved in a research & action pathway (e.g. researchers, policy makers, end-users) to anticipate and make adaptations that increase the likelihood that contributions to action are realized. Despite the fact that the interest in *alignment efforts* is increasing, it remains unclear if and how these efforts ultimately increase the likelihood that research-related contributions are realized. To our knowledge, a method for assessing the relation between *alignment efforts* for new research projects and eventually realized contributions does not exist. The aim of this study is therefore to design a method that can be used to map research-related contributions and relate these contributions to *alignment efforts*. In developing *Contribution Mapping*, we build upon insights from existing methods, such as the Payback framework and enrich this with a perspective on research and utilization that is informed by social studies of science, insights into the evaluation of change in complex open systems and our own experiences with trying to assess research ‘impact’ in various countries [8,15,20,30-32].

This article continues by laying out our perspective on research and knowledge utilization, and categories of research-related contributions. We then explore explanations for research utilization in the literature and identify nine specific *alignment efforts*. This is followed by a description of the stages, steps and procedure of *Contribution Mapping*. In the final section we reflect on the developed method and on the way it could be used to better employ research to contribute to an envisioned future.

### Perspective on utilizing research knowledge

The first step in designing *Contribution Mapping* is to sketch a perspective on how knowledge is produced and utilized and how research-related contributions are realized. The perspective below is inspired especially by social studies of science and actor-network theory [19,31,33-35].

We describe research broadly, as an organized search process in which knowledge is designed [31]. To produce a new knowledge, elements such as local observations, test results, theories, concepts and circumstances, are aligned into a configuration. This process continues with designing a knowledge claim that is to stand in for (and partly blackboxes) the aligned elements [31]. The produced knowledge claim can be added to, and stored in, knowledge reservoirs. These reservoirs can be explicit and contain codified knowledge, such as scientific journals or databases, but can also be more diffuse in nature, such as the knowledge available in a group of people. Knowledge can also be embodied in innovative artefacts, such as an MRI-scanner or new medicine. Knowledge always takes a material form, it can never exist by itself, and work is required to maintain and transport it [19]. It is a configuration that continuously has to be remade.

In the literature on knowledge utilization in the health sector, there has been limited attention for the role of the users of knowledge and the evolving worlds in which they operate. Users may have different interpretations of knowledge and existing arrangements and ongoing change activities in their world may constrain or enable utilization [19,34,36].

The next step in sketching our perspective is therefore to characterize that evolving world. The world has a certain order that sustains itself, without fully determining the future and is at the same time changing and full of ideas for change [37]. That order of the world and the changes that occur are socio-material [35]. The architecture and equipment of a hospital, the design of a wheelchair, the genetic code of a virus, the composition of a vaccine, the infrastructure for reaching patients: they are materialisations of a specific way of doing and embody a specific institutional (or social) code or script [38,39]. In turn this material order structures the practices for health and its societal embedding: social relations between a health worker and a patient, between hospitals, insurance companies and researchers and the wider social environment such as the pharmaceutical industry, government, trade agreements. In such a socio‒material order, physical as well as institutional boundaries are created and maintained that enable some developments and constrain others [35].

While the social and material order sustain each other and provide some continuity, there is also ongoing change, that is full of ideas for change and implementation strategies of a variety of involved actors [37]. A novel treatment that is implemented, scripts in new technologies, evolving viruses, innovative buildings that challenge social codes and new policies, visions and strategies may work in different directions and gain their own kind of momentum. They interact with the existing socio-material order and the embodied scripts, shared stories and visions that provide direction. In this evolving mosaic of socio-material ordering and multiple ongoing change activities, knowledge utilization takes place.

Users and the world in which they operate, play an important role in articulating knowledge and realizing its productivity. Callon provides a perspective to analyze this embedded role [34]. This perspective begins with the idea that actors are constantly formulating and pursuing implicit or explicit scenarios about the future, with the intention of shaping this future. In these actor-scenarios, other people, technologies, knowledges, artifacts and institutions, among other things, are assigned roles as characters in a ‘fictitious script’ about the future [38].

These actor-scenarios depend on what is already present in the world of the actor who ‘narrates’ the scenario and on what he needs. Depending on the forces at play in a scenario and the situation in which a scenario is presented, there can be a particular, sometimes very explicit, need for knowledge. From this action perspective, knowledge can play a role in all kinds of ways. The knowledge that is introduced into a scenario can confirm, support or strengthen elements of an evolving scenario or introduce new elements. Knowledge can also be used to undermine the actor-scenarios of others. Regardless of the role knowledge plays, its use can always be analyzed in terms of evolving actor-scenarios.

Knowledge utilization can be described as incorporating knowledge into an actor-scenario as a means of contributing to its strength and scope. By introducing knowledge from outside, actors can decrease the amount of complexity they have to reduce themselves. Furthermore, it allows them to incorporate things into their scenario that are not present in their own world (e.g. a new innovation, an incidence number or new diagnosis based on controlled observations, scientific status). The introduction of knowledge can strengthen an actor-scenario and enable an actor to deal better with complexity and uncertainty. The addition of new knowledge can increase a scenario’s robustness against attacks from others, but may also make it a bit more complicated.

Research knowledge aims at a certain generality, which makes it interesting and applicable in other cases elsewhere. Utilization, however, is concrete and locally specific. To utilize knowledge and realize a contribution, research knowledge has to be translated. This translation has a cognitive component, which involves translating to a form and content which is applicable, and a social component, which involves translating from the locus of production or storage, to the locus of utilization which has to be rearranged to bring knowledge in [21]. The realization of an actor-scenario requires work, especially when large changes in a local situation are needed. All kinds of efforts may be required to ensure that the actors in a scenario perform their assigned roles. Still, the scenario may unfold differently than originally intended: people may follow their own plans, technologies may not function as expected, a virus may become drug resistant or institutions may resist change. The actors, presented as characters in one scenario, may also make a counter move [34]. To resist the assigned role, they can start telling and initiating an alternative scenario. There may be further reactions, other actors and background resources may be mobilized and further actors may start to tell and pursue actor-scenarios themselves. Ultimately, the scenario-building activities lead to new relationships and action, whereby something actually changes. At the same time, this change is accompanied by constantly new actor-scenarios and their interactions. There will be a net effect with an overall direction of change. Not because there is consensus, but because the elements (actors and links) in the scenarios have become entangled [37,39]. The dynamics lie in the interaction between the actor-scenarios and the significance this has for changing worlds. Together, these scenarios, sometimes explicitly, shape the evolution of our world and the use of knowledge in it.

From this perspective follows that the agency in knowledge utilization is distributed between a number of actors that play a role in a scenario (e.g. the user, the knowledge, other human and non-human actors) and actors in interacting scenarios. Eventual change cannot be attributed to a single source. What we can do is try to analyze the roles played by the actors in the scenario and trace if and how knowledge was brought in and turned into a matter of concern.

### Linked actors and key users

In the study of research and utilization, we would ideally follow all relevant actors, explore all the actor-scenarios put forward and trace all routes that knowledge travels. In reality the dynamics of knowledge utilization and limited resources available for such analyses make this almost impossible. For practical and analytical reasons, we need to prioritize the actors that are followed and utilization processes that are explored.

The actors that are followed are selected in two steps. First, we focus on the actors that shape the research process and the initial route to utilization. This group of actors comprises the investigators and those with whom they interact for the research project, the so-called *linked actors*. Examples of *linked actors* are those asked to give feedback on a research proposal, participants in a research project, practitioners engaged in interpreting the findings or policy makers with whom findings are discussed. Together, the group of investigators and *linked actors* possesses a number of characteristics which makes it both interesting and practical to focus on them. This group includes those who can anticipate utilization during knowledge production and those who are most aware of the results. Another advantage of focusing on *linked actors* is that they can usually be identified.

Within the group of involved and *linked actors*, we subsequently try to identify the most interesting potential users, the so-called potential *key users* (from the perspective of action for health). The identification of potential *key users* requires a certain interpretation of the meaning of the research project, how the results can be used and who the relevant influential actors are in action for health. The selection of *key users* will influence the results of *Contribution Mapping*. The selection process should therefore be transparent. Engaging stakeholders in the mapping process in this selection process may help in making the most appropriate selection and increase the acceptance of the results.

To identify *key users*, we search for actors that take a central place in relevant networks (in action for health) and seem most capable in employing research knowledge to contribute to action for health. In health care, these actors may be influential policymakers, representatives of patient groups, opinion leaders or others who seem capable of creating and realizing influential scenarios in which the produced knowledge may be useful.

The focus in Contribution Mapping is on the potential *key users* among the group of investigators and *linked actors*. Depending on the specific purpose of a mapping exercise, further utilization through other actors who have not been ‘linked’ can also be explored. This again requires the selection of potential key users who will be interviewed. In identifying these actors, a forward approach can be taken, in which the routes of knowledge are traced, by interviewing actors that interacted with the linked actors, for instance. In a backward approach, a specific group of actors is selected (e.g. relevant policymakers at the MOH) and they are interviewed about utilization.

### The three-phase process model

**Figure 1:**
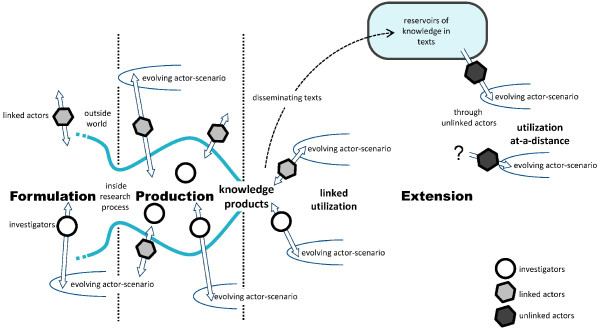
**The three-phase process model.** In the graphical representation of the three-phase process model, the two vertical lines separate the three phases. The search process narrows when a research proposal is formulated. At the beginning of the production phase, the search processes may widen again, before they are narrowed and the knowledge products are realized. During the production phase, there may already be some dissemination and uptake of emerging knowledges. After the knowledge products have been realized, the extension phase commences with dissemination and utilization in evolving actor-scenarios. Investigators are inside the research process, while *linked actors* are outside, but able to interact with the process. Both investigators and *linked actors* can connect the research process to evolving actor-scenarios.

In the second step in developing *Contribution Mapping*, we combine the perspective on research and utilization and the idea of *linked actors* and *key-users* with the existing models in the literature, which results in a three-phase process model (see Figure 1). Each of the three phases in the process model indicates typical activities, which may be linked through more diffuse ‘reservoirs’ at a collective level. The demarcation of the three phases is made for practical purposes. In reality, activities, such as articulating the research question or narrating of actor-scenarios, may temporarily stabilize and continue in other phases. To demarcate between the three phases, we have identified two specific events in the research and contribution pathway.

The first phase is the **formulation phase**. In this phase the activities are generally oriented towards directing search processes and mobilizing resources, and the formulation, selection and funding of specific research projects. Based on promises, expectations and negotiations, dominant ideas emerge about possible research directions and priorities. Formally set research agendas, the commissioning of research and ad-hoc prioritization may involve efforts to align research and action (e.g. when health workers and patients are engaged in priority setting). Researchers may also seek alignment by anticipating perceived needs, or by engaging potential users in the formulation of research proposals. The selection of research proposals and funding of research projects provides another opportunity for those involved to attune research and action. The moment that the investigators become aware that a research project is being funded is used to demarcate the formulation phase from the next phase.

In the **production phase,** the activities take place to realize the knowledge products. This may involve an array of activities. At the research site(s), the preconditions have to be realized to make research possible. This may include training staff, adapting organizational practices, acquiring equipment, establishing relations with policymakers, recruiting participants, etc. These activities are interesting because they can be a first contribution or provide the foundation for later utilization. To produce new knowledge, elements, such as controlled observations, theories, statistical analyses, participants, computer outputs, discussions, etc., are mobilized and aligned into a configuration. Subsequently, a new knowledge claim is designed that is to ‘stand in’ (or ‘speak’) for these aligned elements [28]. The investigators and others involved may learn something during the research process, and the new knowledge may become part of the knowledge they possess.

Even before the formal knowledge outputs are designed, there may be uptake of emerging knowledge in practice, and the new relations and changes made at the research site(s), may contribute to action [7]. The moment that the investigators determine what their findings are is used to demarcate the production phase from the knowledge extension phase.

In the **knowledge extension phase,** the activities are aimed at making knowledge available to potential users and initiating and stimulating utilization. The investigators, *linked actors* and/or others may disseminate the produced knowledge and initiate and stimulate utilization. Through presentations, targeted dissemination and publications in popular media and scientific journals, the new knowledge may be added to diffuse and more explicit reservoirs of knowledge. The various and overlapping knowledge reservoirs are accessible to different potential users in different ways, in terms of their absorptive capacity and competencies for utilization.

Utilization depends on actors that grab knowledge from these reservoirs and combine and use them for their own purposes, in a complex world full of ongoing processes. Investigators*, linked actors* or unknown others may initiate and stimulate the activities which turn a novel combination of knowledges into a going concern as a part of practices, a component in a successful innovation or an element in decisions and their implementation. This is how the promised contributions are actually realized.

### Contribution categories

Based on the above perspective and the three-phase process model, four categories of research-related contributions can be distinguished. The first category pertains to the change in the ability and actions of the investigators and *linked actors* that results from the research activities. The second category contains the knowledge products that are added to reservoirs of codified knowledge and the research domain. The third category contains the contributions to action through utilization of the knowledge produced by the investigators or *linked actors*. The fourth category contains indications of contributions realized through utilization by non-linked actors. Utilization at-a-distance is separated from linked utilization because the former is often much more difficult to assess.

#### Change in abilities and actions of involved and *linked actors*

This category comprises changes in the investigators and *linked actors* that are related to the *activities* of research. The *activities* that research comprises may lead to new competences, behaviours and relationships among the involved and *linked actors*. Research *activities* may also lead to the introduction of new practices, protocols and the identification or awareness of problems at the research site.

#### Contributed knowledge products

This category comprises the realized knowledge products that are added to reservoirs of codified knowledge and to the domain of research. This includes scientific publications, publications in other media and new research projects, protocols, methods and equipment (e.g. publications, new research methods, better targeting of future research).

#### Contributions through linked utilization

This category comprises contributions to action, through utilization of the produced knowledge by the investigators or *linked actors*. The produced knowledge is added to an evolving actor-scenario that is at least partly enacted. The new knowledge becomes part of a novel combination that is turned into a going concern as a part of practices, a component in successful innovation or as an element in decisions and their implementation.

#### Indications of utilization at-a-distance

This category comprises contributions to action through utilization of the knowledge produced, by actors that are not involved in, or linked to, the research project. These contributions are described tentatively as indications of utilization at-a-distance as they may be difficult to identify and triangulate.

### Deriving *alignment efforts* from explanations for research utilization

The next step in the development of our method is identifying specific efforts during research formulation, production and extension which aim to enhance the contribution of research to action. These so-called *alignment efforts* are defined as deliberate efforts aimed to increase the likelihood that a contribution to action is realized. By assessing both the presence of *alignment efforts* and the realized contribution to action for each research project, we can analyze the extent to which *alignment efforts* are related to specific contributions (e.g. engaging policymakers in proposal formulation increases the chance that research is used in policy making).

To identify a set of interesting *alignment efforts*, we searched extensively for publications of empirical studies that 1) take research processes as a starting point, 2) simultaneously analyze various *alignment efforts* and 3) relate these to utilization. While we found several studies that focus on characteristics of the users of research and their context [22,40-46], the characteristics of research products and interactions between stakeholders [45,47-53], we found no studies that matched the three search criteria. We did find three studies in which various explanations for research utilization were simultaneously considered [42,43,45]. These three studies use a similar theoretical framework that describes two categories of explanations of research utilization: engineering explanations and socio-organizational explanations. In these frameworks, the latter category is divided into three sub-categories: organizational interest explanations, ‘two communities’ explanations and interaction explanations. We have chosen to build upon these studies because they provide a detailed theoretical foundation and an empirical example.

### Engineering explanations

Pioneering studies aiming to explain research utilization focused on so-called engineering explanations, i.e. the technical merits (or instrumental value) of the knowledge as the key for utilization [47,48,54-56]. Landry and colleagues divide these engineering explanations as focusing on two different clusters of dimensions: research findings (validity, content attributes, compatibility, complexity, observability, trialability, reliability, divisibility, applicability, radicalness) [57-59] and the type of research (quantitative, qualitative, basic, theoretical, applied, research domains and disciplines, etc.) [60,61]. While there is prima facie plausibility that such factors will have effect on utilization, several studies have shown that these engineering explanations have limited explanatory power [40,62,63]. A reason for this might be the indeterminate directional influence of these factors. Quantitative studies may be influential because the findings seem more concrete, but may also be too technical and provide limited direction to policymakers, for instance. Because past operationalizations of the engineering explanations have led to ambiguous predictions and results, their usefulness in exploring the contribution of research to action seems limited.

### Socio-organizational models

The socio-organizational models of research utilization focus on the processes and interactions during a research project and the relations with context, instead of the intrinsic atomistic characteristics of research. These models can be further divided into three – partly overlapping – subcategories that emphasize different aspects: 1) organizational interest, 2) ‘two communities’ and 3) interactions.

The organizational interest explanation predicts that the *needs* of organizations and *features* of organizations (e.g. policy domain, organizational structure and role, positions) shape how actors utilize research, with the corollary that the utilization of research increases if it is oriented to the needs of end-users [64]. Empirical studies have shown that the use of research increases as users consider research pertinent, as research coincides with the users’ needs, as the users’ attitude is to give credibility to research and as results reach users at the right time [43,45,61,65]. To orient a research project to the needs of users, these users have to be identified and their needs have to be articulated. One way of doing this is engaging potential users in setting research priorities [66-68]. These priorities then have to be taken into account, during the formulation and selection of research proposals. Research can also be directly linked to a question of a specific organization, up to being commissioned by it. An advantage of directly linking research is that the specifics of a question can be taken into account including the framing of the question and specific windows of opportunity in the decision-making context [69]. Another direct way to link research to action is when a person involved in policy or practice is involved as one of the investigators (e.g. technical advisor, board member) [70]. Actors with this kind of a double role can attune the research project to needs in the action domain, but also prepare the ground for utilization in the action. The potential of such a double-role actor depends on his capability to create alignment between research and action.

The ‘two communities’ explanation predicts that the cultural difference between the research community and the policy community is the main reason for low levels of research utilization [49,71]. This explanation has a long history and originally focused on differences in norms and values and language of communication between science and policy. More recently, other aspects have also been grouped under this explanation [72]. While there are real differences between the domains of research and policymaking, detailed investigations of research and policy have rendered the distinction between these two communities less meaningful. Viewed up close, science turns out to look a lot like other social institutions, full of norms, beliefs, ideologies, practices, networks and power and deeply engaged in the production and management of social order [73]. Similarly, policymaking processes rely deeply on the production of matters of fact to acquire and retain legitimacy. In addition, there is the tremendous diversity within the research (applied social science, quantum physics) and policy (formal legislation, technical guidelines) domain. For our method we are not just interested in the domains of ‘research’ and ‘policy’, but also in the ‘health’ domain (which can also be policy actions). An additional insight is that policy is not made in one place at the apex of society (or at the top of the research and health systems), but at various levels, including by health practitioners who function as ‘street level bureaucrats’ [74]. Instead of dealing with two communities, we have to deal with three partly overlapping and stratified domains. Efforts to deal with the differences between these domains traditionally focus on literally translating research results to a format and language that is deemed suitable for policy and practice, providing more context specific recommendations and more targeted disseminating to potential *key users* in action.

Most recently, the explanations of research utilization focus on the interaction between researchers and the users of their knowledge products [22-24]. The interaction explanation predicts that the utilization of knowledge depends on the various interactions occurring between researchers and users. Interactions can lead to trust, mutual learning and the anticipation of utilization on both the research and action side. The effect of interactions depends on the capability of actors to align research and action and on the phase that a research project is in. Interactions during proposal formulation may lead to adaptations of the study design, while interaction during the interpretation of results may help to frame them in a decision-making context.

### Analyzing *alignment efforts*

In the previous section on explaining research utilization, various *alignment efforts* are described, such as engaging potential users in setting research priorities, formulating research and interpreting results, employing *double-role actors* in research and disseminating research results to potential *key users*. Depending on the aim of the mapping exercise, involved actors may prospectively select specific *alignment efforts* on which to focus the analysis. Another approach is to try to retrospectively analyze which *alignment efforts* have played a role in realizing contributions. *Alignment efforts* may have a combined and context-dependent effect and different aims may require different efforts. In-depth case studies may be especially useful in analyzing how *alignment efforts* enhance contributions in context. It may also be interesting to conduct multiple case studies and search for patterns in the relation between *alignment efforts* and contributions.

In Table 1 nine *alignment efforts* are described as examples. The presence and functioning of these efforts can be explored for each assessed research project. When a large number of projects is assessed, ordinal scales can be developed to score the presence of each *alignment effort* in the mapped projects. These scores can then be used for the identification of patterns in the relation between *alignment efforts* and realized contributions.

**Table 1:** Example of nine alignment efforts

**1**	**Attuning research to formally established research priorities**
	This effort may be of interest if there is a formally established list of research priorities that is intended to attune research to needs of end-users (e.g. patients, policymakers, health workers). When the needs of stakeholders from the action side are taken into account in priority setting, this provides a first step to towards attuning research to action. Investigators then have to take these priorities into account when formulating research proposals and these priorities have to play a role when selecting projects for funding. Assessing the role of this alignment effort, allows these various steps and/or the overall correspondence between a research question and the set priorities to be explored.
**2**	**Attuning research to action processes in which investigators have a role**
	In this *alignment effort*, one of the investigators has a double role in action and may be considered a potential *key user*. Investigators may be involved in action processes as an adviser or board member, but also as the director of health services. Such double roles may be ideal for attuning research to policy processes. When assessing this effort, the focus can be on the extent to which research is attuned to needs and/or on the capability of the *double-role actor* to influence the dynamics in both research and action.
**3**	**Engaging potential*key users*in research formulation**
	Engaging potential *key users* in the formulation of research may help the investigators to better understand the needs and expectations from action and may help them anticipate the context in which knowledge may be used. Engagement may help potential *key-users* anticipate utilization on the action side. When assessing this effort, the focus can be on the adaptations made to a research proposal as a result of this engagement, anticipations through engaged actors on the action-side, changes in relations between actors (e.g. increasing trust and understanding) and on the role and capability of the engaged actor(s) in influencing the dynamics in action.
**4**	**Engaging potential*key users*during the production phase**
	Engaging potential *key users* in the conduct of research may help the investigators better understand the needs and expectations from action and help them anticipate the context in which knowledge may be used. On the other side, engagement may help potential *key users* better understand the research and anticipate utilization. Engagement may lead to new relations and provide a foundation for later utilization in action. When assessing this effort, the focus can be on the adaptations made to the research project as a result of this engagement, anticipations through the engaged actors on the action-side, changing relations between actors (e.g. increasing trust and understanding) and the role and capability of the engaged actor(s) in influencing the dynamics in action.
**5**	**Engaging potential*key users*in interpreting the produced knowledge**
	Engaging potential *key users* in interpreting the produced knowledge may help frame the results in the context in which they may be used and help potential users to articulate actor-scenarios. In addition, personal interaction makes it possible to build trust and exchange knowledge with potential *key users*. When assessing this effort, the focus can be on the framing and interpretation of the results, the changing relations between actors, the exchange of knowledge and on the role and capability of the engaged actor(s) in influencing the dynamics in action.
**6**	**Dissemination targeting potential*key users***
	This alignment effort comprises one-way dissemination of knowledge products (texts) targeted towards potential *key users*. When assessing this effort, the focus could be on the extent to which knowledge products are adapted to specific audiences, the extent to which dissemination specifically targets potential *key users* and the way these potential *key users* receive and interpret the sent texts.
**7**	**Utilization efforts by investigators**
	In this *alignment effort*, one of the investigators takes on the role of *key user*. Such a *double-role actor* may be involved in policy processes as adviser or board member, but also as an influential actor with the authority to make decisions in which the new knowledge is used. These *double-role actors* may initiate actor-scenarios in which the new knowledge has a role and actively stimulate the realization of these scenarios. When assessing this effort, the focus can be on the capability of the *double-role actor* to initiate actor-scenarios, bring new knowledge in and stimulate the realization of these scenarios.
**8**	**Utilization efforts by*linked actors***
	In this *alignment effort*, a *linked actor* takes on the role of *key user*. Through their role or formal function in action, the *linked actor* may initiate utilization by creating actor-scenarios with a role for the new knowledge and stimulating the realization of these scenarios. When assessing this effort, the focus can be on the capability of the *linked actor* to initiate utilization, bring new knowledge into actor-scenarios and stimulate the realization of these scenarios.
**9**	**Utilization efforts by non-linked actors**
	In this *alignment effort* an actor who is not linked to a research project is expected to take on the role of *key user* in utilization at-a-distance. A non-linked actor can become a *key user* by taking codified knowledge from a reservoir and using it to initiate and realize actor-scenarios. Assessing this effort may be difficult as the non-linked actors that use the results have to be identified. The focus of assessment can be on the extent to which non-linked actors have access to the knowledge reservoirs, their absorptive capacity and their capabilities for utilization.

## Contribution mapping

In the previous sections we have described 1) an explicit perspective on research and utilization, 2) the three-phase process model, 3) categories of research-related contributions and 4) explanations for research utilization and related *alignment efforts*. In this section we describe the structure and procedures of *Contribution Mapping*.

### Stages in *contribution mapping*

**Figure 2:**
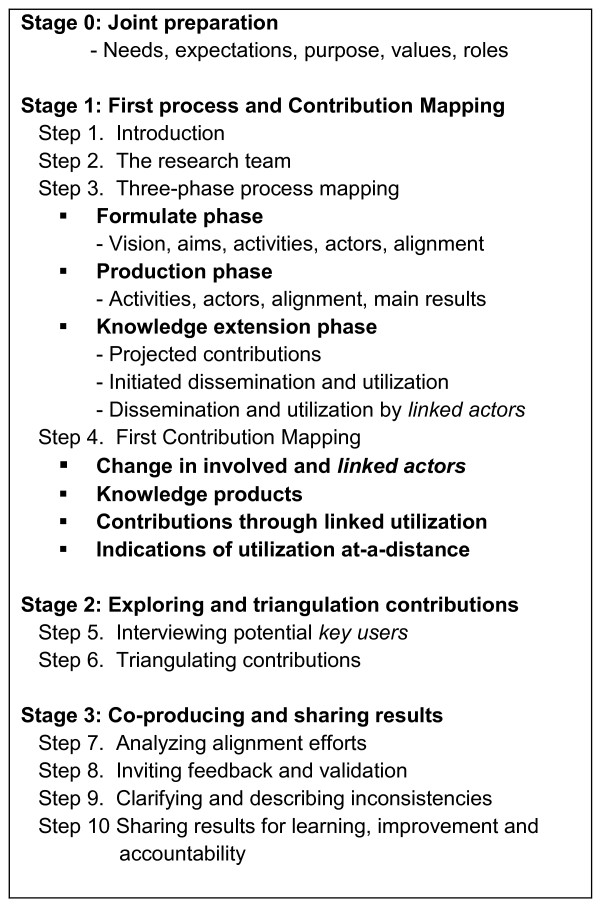
**Stages and steps in*Contribution Mapping.***

There are four stages and ten steps to *Contribution Mapping* (see Figure 2). For each research project that is assessed, all ten steps are followed and both a process map and a contribution map are developed. In the first stage of *Contribution Mapping* (step 1-4), the investigators of a research project are interviewed to start developing a process map. The process map is iteratively developed throughout *Contribution Mapping* and includes the main activities for the three phases, the *linked actors* and potential *key users*. The first stage ends with a first estimation of the contributions, as perceived by the investigators, which are added to the contribution map. In the second stage, potential *key users* and other informants are interviewed in order to trace, explore and triangulate possible contributions. In the third stage, the *alignment efforts* are analyzed. Preliminary results are shared with the stakeholders for feedback and validation. After inconsistencies are clarified or described, the results are shared with stakeholders for learning, improvement and accountability purposes.

The general interview strategy in *Contribution Mapping* is to start with asking open questions and continue by probing and providing more specific examples.

### Stage 0: Joint preparation

#### Needs, expectations, purpose, values, roles

The aim of step one is to explain the process of *Contribution Mapping* and jointly clarify needs and expectations, the purpose of conducting *Contribution Mapping* and the values and roles of those involved. Why is *Contribution Mapping* employed, and what is expected from it? What is the rationale that connects the mapping of research contributions to some higher purpose? Who are involved and who are expected to learn or benefit from the results? In what context is the assessment conducted? *Contribution Mapping* may be employed for various purposes, such as accountability to investors, learning how to better employ research and improving the extent to which beneficial contributions are realized. Table 2 provides an indication of how the approach can be attuned for each purpose. Depending on the purpose, specific *alignment efforts* may be selected, to focus on during later steps.

**Table 2:** **Using*Contribution Mapping*for specific purposes**

**for:**	**Accountability**
	When using *Contribution Mapping* for accountability purposes, it should be clarified who should be accountable to whom (researchers to funders, funders to taxpayers, etc). Assessments for accountability purposes tend to focus on outcomes, combined with inputs and with the effects of exogenous factors. While it may be interesting to show contributions to policy, practice and innovation to the outside world, this may also lead to unrealistic claims and expectations. If accountability is the aim, the focus may well be on the activities for which researchers can be held accountable, such as initiated dissemination, and *alignment efforts,* such as engaging potential end-users. In addition, outputs and contributions can be described.
**for:**	**Learning**
	When using *Contribution Mapping* for learning purposes, the key questions are why research-related contributions are realized and how these contributions relate to research activities (in context). Depending on the precise question, multiple cases can be compared, or the focus may be on unique deviating cases. Cross-case analyses can be useful for revealing patterns between *alignment efforts* and contributions. Analyzing specific cases that deviate from the expected may help deepen our knowledge of how and why research contributes to action, in context. Analyzing multiple cases can also be an interesting test for the functioning of research programmes or systems.
**for:**	**Improvement**
	When using *Contribution Mapping* for improvement purposes, the key question is who should do what differently to improve performance? This requires inside-the-black-box relationships that connect changes in processes and activities to changes in contributions. To achieve improvement, a single assessment may be insufficient. Improvement generally requires trying out something new, careful observation and continuous learning in a conducive environment. The process and contribution map and analyses of *alignment efforts* can be used as a monitoring tool in the improvement process.

In *Contribution Mapping*, we do not assume that evaluation is value-free. The process is intended to be participatory and stakeholders are invited to bring their ideas, perceptions, frames and values into the mapping exercise and take on an active role. The roles and functions of those involved in the evaluation are not predetermined and should therefore be clarified at this stage. Depending on the situation and purpose of *Contribution Mapping*, a useful division of tasks and responsibilities has to be decided on.

### Stage 1: First process and contribution mapping (step 1-4)

The main activity of stage one is interviewing the investigator(s) of the research project that is assessed. This interview is best held with the principal investigator and, if possible, another investigator involved. This stage ends with a first estimation of research-related contributions, which is based on the perceptions of the investigators.

### Step 1. Introduction

The aim of step one is to introduce *Contribution Mapping* to the investigator(s) that are interviewed and lay out the three-phase process map. To prepare for the process mapping, documents, such as research proposals, reports and publications, are read. The interview with the investigator(s) starts with explaining the purpose and stages of *Contribution Mapping* and structure of the interview. After the emphasis on actors and processes is explained, the three-phase process map is introduced (formulation, production, extension). A timeline is drawn and the two demarcation moments are identified and added to the timeline.

### Step 2. The research team

The aim of step two is to map and characterize the research team and identify potential *key users* amongst them. The research team consists of the enlisted investigators and others professionally involved in the study (e.g. research assistants, health workers). For each investigator, age, background, research and policy experience, roles in the project (in what activities involved) and formal and informal roles in relevant decision-making processes are described (e.g. in advisory committees, policy processes). This information provides insight into how the investigators may link, through their past and current functions, roles and actions, the research process to relevant decision-making processes and thereby enables the identification of potential *key users.* Next, the others who are professionally involved in the study are described (e.g. manager of involved institute, nurses, medical doctors, research assistants). If it appears relevant, further questions are asked to establish how they were involved and if they are likely to function as *key user*.

### Step 3. Three-phase process mapping

The aim of step three is to start filling in the process map, from the perspective of the investigators. For each of the three phases (formulation, production, extension), the main activities, *linked actors* and potential *key users,* as well as *alignment efforts,* are identified. (As the focus is generally on linked utilization, the *key users* among the investigators and *linked actors* are selected first. Second, other *key users* can be identified to explore utilization at-a-distance).

### Formulation phase

#### Vision, aim, activities, actors, alignment

First, the vision underpinning the research project is explored. Why did the investigators initiate this research project? (e.g. to inform policymaking, publish high impact articles, contribute to specific changes). After clarifying the vision, the next step is exploring how the research project aimed to contribute to this. What was the aim of the research project?

The origin and formulation of the project is explored. Where did the idea for the research question come from? Who was involved in formulating the research proposal? Who were considered potential *key users*? Was the proposal discussed with potential users? If so, these are added to the process map as *linked actors*, and the role and influence of these individuals is explored to determine if they are potential *key users*.

The influence of interactions with potential users is explored. Was the research proposal adapted as a result of interactions? Did the potential users initiate any actions because of awareness of the research formulation (e.g. postpone decision-making)? Finally, specific *alignment efforts* can be further explored (e.g. Was the proposal attuned to a priority of a research agenda?).

### Production phase

#### Activities, actors, alignment, main results

The process mapping continues with the production phase. Again, the aim is to get an overview of the main activities, *linked actors* and potential *key users*, as well as the selected or unexpected *alignment efforts*. What were the main activities and who was involved? Have new actors been linked to the research process through engagement or interaction? If so, the role and influence of new *linked actors* is explored to identify potential *key users* amongst them. The influence of such interactions is explored to determine if this has led to new alignments. Were any adaptations made to the research process as a result of these interactions?

The mapping of the production phase ends with a description on the main results. The investigators are asked to describe the main results with minimal interpretation, as they would in the result section of an article.

### Knowledge extension phase

#### Contributions expected by the investigator

The exploration of the extension phase starts with asking the investigators to describe the *meaning* and *consequences* of the main results for policy, practice and health. How should the results be picked up and utilized, and who should take up this role? The investigators are thus asked to sketch their actor-scenario in which the produced knowledge has a role and *key-users* are identified.

#### Initiated dissemination and utilization

Extension efforts may include one-way dissemination, but also more interactive efforts to initiate and stimulate utilization.

First, the interpretation of the results is explored. Have any previously *linked actors* been engaged in interpreting and framing the results? Have new actors been engaged in interpretation and thus become linked to the research project? Are there any potential *key users* among them? Second, the dissemination is explored. How and when were the results disseminated? Did this involve interaction that has led to new *linked actors* and potential *key users*? Were the results published in scientific journals, popular media and/or on the internet? Has the data been made available for use by others?

Third, the utilization that is initiated by the investigators is explored. Did the investigators try to use the results themselves in action processes? They might have a function in action (e.g. a health director conducting research) or take on such a role and start to tell and create new actor-scenarios. Did the investigators engage new actors to initiate utilization? (These newly engaged actors become *linked actors*). Where there any potential *key users* among them?

#### Dissemination and utilization by *linked actors*

In a similar way, a first exploration is made of extension through the *linked actors,* especially the potential *key users*. Are the investigators aware of any efforts by *linked actors* to further disseminate the results or initiate utilization?

At the end of step three, a first process map has been made around a timeline, with for each phase a summary of the main activities, the *linked actors*, potential *key users* and the *alignment efforts*. This map provides a transparent overview that can be further explored, discussed, added to, modified and triangulated.

### Step 4. First contribution mapping

The aim of step four is to start identifying possible research-related contributions and tentatively add them to the contribution map. We begin this process by asking the investigators about the realization of contributions in which they were involved. We then ask the investigators to indicate routes towards contributions through *linked actors* and possible others. Each of the four contribution categories is briefly explained to the interviewees, after which the perceived contributions are explored and described one by one.

#### Change in involved and *linked actors*

The first contribution category comprises changes in the involved and *linked actors* that are related to the *activities* of research. First, research-related changes in the investigators are explored. Have they developed new skills, competences and relations as a result of the research activities? What have they learned and has this changed their behaviours and actions? Subsequently, similar questions are asked regarding the other involved or *linked actors* or groups (e.g. research assistants, health workers, participants, policy makers).

Are the investigators aware of changes in competences, behaviours, relationships and actions in any these actors or groups? This provides an indication of changes in these other actors.

#### Knowledge products

The second contribution category comprises the knowledge products that are added to reservoirs of codified knowledge and the domain of research. Based upon the described dissemination activities, the investigators are asked to which reservoirs the produced knowledge has been added. This may include publication of the results in scientific journals, the local media, internet, etc., but also research data that is made available to others. Finally, the investigators are asked to describe other contributions to the research domain, such as methods that are used by others, better targeting of new research, newly funded research projects, etc.

#### Contributions through linked utilization

The third category comprises contributions through linked utilization, which refers to utilization of the produced knowledge through the investigators or *linked actors*.

The investigators are asked to describe if and how the produced knowledge has been used to contribute to action, and by whom. Interviewees are asked what role the new knowledge played in evolving actor-scenarios. Some interviewees may have the tendency to overestimate the contributions, while others may downplay the use or role of the results. To increase the reliability of the responses, the interviewees are encouraged to describe all the potential contributions. The process map, which includes the potential *key users,* is used to identify potential contributions (e.g. Has this policymaker that was engaged in interpretation used the results?). These contributions are explored one by one through a critical dialogue, in which the process map is used to relate them to specific actors, events, activities, *alignment efforts* and other ongoing processes. This provides an overview of the utilization of the results by the *linked actors*, as perceived by the investigators.

#### Indications of utilization at-a-distance

The fourth contribution category is utilization by actors that have not been involved in, or have not interacted with, those involved in the research project. This utilization at-a-distance results from external actors that take codified knowledge from a reservoir, without interaction with the investigators. Utilization at-a-distance can only be described if one of the investigators, *linked actors* or other stakeholders are aware of it and describe it to those involved in *Contribution Mapping*, or when knowledge used in texts or artefacts can be traced back to a research project. Utilization at-a-distance is not the focus of *Contribution Mapping*, as it is difficult to describe, trace and triangulate. Depending on the goals of those involved, indications of utilization at-a-distance can be explored and described.

### Stage 2: Exploring and triangulating contributions (step 5-6)

In the second stage of *Contribution Mapping*, the main activity is interviewing potential *key-users* and other relevant informants to further explore and triangulate utilization. We focus below on contributions through linked utilization, but step five to ten are similar if the focus is on other contribution categories.

### Step 5. Interviewing potential *key users*

The aim of step five is to explore and, if possible, triangulate contributions among potential *key users*. Based on the process map and described contributions, a selection is made of the most interesting potential *key users,* and they are approached for an interview. The interview starts with questions about their characteristics (background, experiences in research and policy, formal and informal roles in related decision-making) and continues with their interactions with the research project and awareness and interpretation of the results. Next, they are asked if, how and why they have been involved in utilization. Interviewees are asked how new knowledge was brought into evolving scenarios and what role it played. Utilization is further explored through a critical dialogue in which an attempt is made to trace pathways to specific actions, times and places. The utilization that others have described (in step 4 or 5) is then shared and the interviewee is asked to give his or her perception of this. If it seems useful, the *key user* is asked to identify others who may further describe if, how and why results were used in evolving scenarios.

### Step 6. Triangulating contributions

The aim of step six is to further explore utilization and, if possible, triangulate claims about contributions. Key informants are selected based upon their knowledge about the scenarios in which results may have been used (these key informant may be others involved or *linked actors*). The interview starts with the characteristics of these informants (background, experiences in research and policy, formal and informal roles in related decision-making) and continues by exploring the actor-scenarios in which knowledges may have been used. Next, these informants are asked to provide their perception of utilization. Have the results been used and what role did they play in the evolving scenarios? Claims are explored through a critical dialogue in which an attempt is made to trace pathways to specific times and places.

### Stage 3: Co-producing and sharing results (step 7-10)

In the third stage, the main activities are analyzing the *alignment efforts*, asking for feedback, clarifying inconsistencies and sharing the resulting maps for learning, improvement and accountability.

### Step 7. Analyzing *alignment efforts*

The aim of step seven is to analyze the alignment efforts. The key questions are to what extent which *alignment efforts* were employed and how their functioning relates to the contributions that are realized. If specific *alignment efforts* have been identified beforehand, the information gathered about these efforts can be used to describe their functioning for each research project. A different approach is to start with analyzing the process and contribution map and to identify deductively which *alignment efforts* have played a role in realizing contributions. To analyze how *alignment efforts* relate to contributions, both detailed in-depth analyses of single research projects and comparative multiple-cases studies can be useful.

### Step 8. Inviting feedback and validation

The aim of step eight is to ask interviewees and other stakeholders to provide feedback on the process and contribution maps and the descriptions or scores of the *alignment efforts*.

The preliminary results are shared with the stakeholders (in writing or presented to them) and they are asked if the results are consistent with their perceptions. This is important for validation of the results and enhancing ownership among stakeholders.

### Step 9. Clarifying and describing inconsistencies

The aim of step nine is to clarify and describe remaining inconsistencies. In *Contribution Mapping*, results are not considered to be value neutral ‘facts’. The described contributions are the result of articulating and negotiating different versions of reality. Actors may have different, incommensurable versions of the extent and way knowledge has been used. Inconsistencies are shared with those involved and they are invited to comment. Further clarification is stimulated by asking questions, pointing to blanks in narratives and facilitating constructive discussion. In some cases, inconsistencies may be clarified and a shared story is realized. In other cases, divergent versions of reality remain to exist. This is not unusual and can be expected especially for complex, diffuse and contested utilization processes in which many actors are involved. As an output, the different versions can be described with a comment that a shared version could not be established.

### Step 10. Sharing results for learning, improvement and accountability

The aim of step ten is to share and employ the results for learning, improvement and/or accountability purposes. The information gathered during the first nine steps provides an overview of what has been done and gives a good indication of the contributions that have been realized and the roles of selected *alignment efforts*. The resulting maps should not be understood as fait accompli. The maps provide a time-bound overview of how processes have developed over time and the contributions that are realized at a certain moment. As the world continues to evolve, modifications to the maps can be made.

The way the resulting maps are used depends on the purpose of *Contribution Mapping* (see Table 2). For accountability purposes, the key outcomes, inputs and external factors are identified and shared. For learning purposes, the key lessons are identified by analyzing single cases or comparing multiple cases. If *Contribution Mapping* is employed for improvement purposes, the results could inform the formulation and execution of plans for improvement.

## Discussion

In this paper we described *Contribution Mapping*, a novel approach to research monitoring and evaluation. At a time of growing emphasis on the use of research results and accountability, it is important to map research-related contributions and find ways to enhance the likelihood of beneficial contributions. We have tried to develop a realistic and practical approach that can be used to establish accountability and contains an explicit strategy for learning how the likelihood of beneficial contributions can be enhanced. We hope this makes *Contribution Mapping* a useful evaluation tool for those who seek to better employ research and knowledge to contribute to health, equity and development.

*Contribution Mapping* builds on an explicit description of the process of knowledge production and utilization with an active role of the user, in an evolving socio-material order full of ongoing change activities. This monistic perspective and the practical procedures of *Contribution Mapping* may also be useful for other analyses of knowledge utilization, such as knowledge translation platforms [29].

In the introduction we described a number of challenges and problems that existing approaches for assessing research ‘impact’ struggle with. While recognizing the limitations posed by some of these problems, we have tried to develop a method that is useful in practice. Research utilization depends on distributed agency, which makes it difficult to ‘measure’ and attribute impacts. *Contribution Mapping* assumes that a plausible description of eventual contributions can be realized through a combination of a structured approach, a transparent process and engagement of those involved. In addition, the approach is intended to stimulate learning and reflection by those involved and stimulate further efforts to enhance the contribution of research.

An important choice in *Contribution Mapping* is to focus on the change in the abilities and actions of involved and *linked actors* as well as linked utilization, which is distinguished from utilization at-a-distance. An advantage of focusing on linked utilization, in combination with the *alignment efforts*, is that it directs the attention to what researchers and others can do to enhance the realization of beneficial contributions. This is essential in a learning-based and use-driven evaluation approach. Furthermore, the demarcation of linked utilization provides a certain boundary in ongoing and seemingly endless utilization processes, which is useful when the aim is to analyze and compare a number of research projects.

The initial focus on linked utilization has as a downside that the ultimate contribution to health at the patient level remains beyond the reach of the analysis. If such utilization at-a-distance is of specific interest, the stepwise approach of following actors and the routes of knowledge can be used to further map the pathways to more distant contributions. Retrospectively mapping such long and complex processes may be very difficult. The further the analysis moves away from the research project, the weaker the relation with the research project becomes and the more the attribution problem, user-identification challenge and pathway diversity and diffuseness problem may hamper the analysis. While the focus on linked utilization has its downsides, it makes a useful form of research monitoring and evaluation possible.

The indicators currently used to evaluate research and motivate researchers, such as publications in high impact journals, are unsatisfactory if research is to contribute to better action for health. Counting publications and citations keeps track of how often the ball is kicked across the middle line instead of in the goal. Scoring, in terms of contributing to better action for health, requires a collective effort which can not be attributed to a single actor or project.

Instead of using poor indicators and incentives, or trying to make unrealistic and useless attributions, the focus should be on enhancing the contribution of research to the collective performance. With that in mind, we designed *Contribution Mapping*. The method is intended to reveal how to better anticipate, learn, communicate and align efforts to ultimately increase the likelihood that a contribution is made to collective achievements. We expect the method will be a useful tool for learning and improvement purposes and will allow those involved in research and utilization to take responsibility for the actions within their reach.

An approach to map the utilization of knowledge is much needed. We expect that applying *Contribution Mapping* in practice will provide important insights that can be used to further develop the approach as well as lessons on how research can better be employed to contribute to action for health. An important next step is to further develop a version of *Contribution Mapping* that can be integrated at the planning stage of research programs and projects and assist those involved with employing research to better contribute to an envisioned future.

## Competing interests

The authors declare that they have no competing interests.

## Authors’ contributions

MK developed the theoretical framework, designed the methods and drafted a first version of the manuscript. JS commented on the framework, methods and manuscript. Both authors read and approved the final manuscript.
